# Modeling steady state SO_2_-dependent changes in capillary ATP concentration using novel O_2_ micro-delivery methods

**DOI:** 10.3389/fphys.2013.00260

**Published:** 2013-09-24

**Authors:** Nour W. Ghonaim, Graham M. Fraser, Christopher G. Ellis, Jun Yang, Daniel Goldman

**Affiliations:** ^1^Department of Biomedical Engineering Graduate Program, Western UniversityLondon, ON, Canada; ^2^Department of Medical Biophysics, Western UniversityLondon, ON, Canada; ^3^Department of Mechanical and Materials Engineering, Western UniversityLondon, ON, Canada

**Keywords:** adenosine triphosphate (ATP), microcirculation, capillaries, computational model, simulation, local PO_2_ perturbation, O_2_ regulation, micro-delivery device

## Abstract

Adenosine triphosphate (ATP) is known to be released from the erythrocyte in an oxygen (O_2_) dependent manner. Since ATP is a potent vasodilator, it is proposed to be a key regulator in the pathway that mediates micro-vascular response to varying tissue O_2_ demand. We propose that ATP signaling mainly originates in the capillaries due to the relatively long erythrocyte transit times in the capillary and the short ATP diffusion distance to the electrically coupled endothelium. We have developed a computational model to investigate the effect of delivering or removing O_2_ to limited areas at the surface of a tissue with an idealized parallel capillary array on total ATP concentration. Simulations were conducted when exposing full surface to perturbations in tissue O_2_ tension (PO_2_) or locally using a circular micro-outlet (~100 μm in diameter), a square micro-slit (200 × 200 μm), or a rectangular micro-slit (1000 μm wide × 200 μm long). Results indicated the rectangular micro-slit has the optimal dimensions for altering hemoglobin saturations (SO_2_) in sufficient number capillaries to generate effective changes in total [ATP]. This suggests a threshold for the minimum number of capillaries that need to be stimulated *in vivo* by imposed tissue hypoxia to induce a conducted micro-vascular response. SO_2_ and corresponding [ATP] changes were also modeled in a terminal arteriole (9 μm in diameter) that replaces 4 surface capillaries in the idealized network geometry. Based on the results, the contribution of terminal arterioles to the net change in [ATP] in the micro-vascular network is minimal although they would participate as O_2_ sources thus influencing the O_2_ distribution. The modeling data presented here provide important insights into designing a novel micro-delivery device for studying micro-vascular O_2_ regulation in the capillaries *in vivo*.

## Introduction

The microcirculation plays the important role of delivering and regulating the exchange of oxygen (O_2_) and nutrients to surrounding live metabolic tissue. The transport processes in the microcirculation are tightly controlled and highly integrated. Since proper O_2_ supply to tissue is critical for cellular function and survival, the mechanisms underlying O_2_ transport and distribution have been under thorough investigation. The microvasculature has to continuously adjust erythrocyte distribution and hence O_2_ supply to meet the varying demand of metabolic tissue. During exercise, erythrocyte supply rate increases delivering more O_2_ carrying erythrocytes to the microvasculature. The highly regulated system implies the presence of signaling components that link tissue O_2_ demand with blood flow and microvascular function.

A great amount of evidence suggests the involvement of the erythrocyte as a sensor and a key player in this regulation mechanism (Stein and Ellsworth, 1993; Ellsworth et al., 1995, 2008). Erythrocytes are the carriers of O_2_, bound to hemoglobin, in the microcirculation. Erythrocytes also contain large amounts of adenosine triphosphate (ATP) (Miseta et al., 1993), a potent vasodilator, and are known to release it under hypoxic conditions (Bergfeld and Forrester, 1992; Jagger et al., 2001; González-Alonso et al., 2002). Once ATP is released, it binds to purinergic receptors (P2Y) on the vascular endothelium eliciting a vaso-dilatory signal which is conducted upstream in the arteriolar tree (Ellsworth et al., 2008). The resulting vaso-relaxation of smooth muscle cells (SMCs) surrounding upstream arterioles increases erythrocyte supply rate to meet the metabolic demand of the hypoxic region downstream that initiated the release of ATP from erythrocytes.

For a long time, arterioles have been investigated as a major site of microvascular signaling (Duling and Berne, 1970; Duling, 1974; Jackson, 1987). This has been assumed, mainly, due to the large longitudinal PO_2_ gradients that exist at the arteriolar level. In terms of ATP mediated signaling, the presence of SMCs implies that the released ATP will act locally and instantaneously elicit a signal. However, the relatively short erythrocyte transit times in arterioles are anticipated to largely compromise the localization of this ATP signal, while the parabolic flow profile in the arteriole means only those cells closest to the wall experience the largest change in O_2_ saturation (SO_2_) and hence contribute to the signal. Cells flowing in the centerline will be experiencing a lesser drop in SO_2_ and any released ATP will be carried downstream (Ellis et al., 2012).

Venules may also be involved in the regulation of O_2_ supply since they act as the collectors of large populations of deoxygenated ATP-releasing erythrocytes. However, the diversity in the erythrocyte SO_2_ levels as they drain from various upstream capillaries indicates that venules may only contribute to the overall vaso-dilatory signal (Ellis et al., 2012). Fine-tune regulation of O_2_ distribution to specific capillaries or microvascular units in the microcirculation demands the signal be highly localized. This may only be achieved at the capillary level. Erythrocytes traverse capillaries with long transit times and are in almost direct contact with the capillary endothelium. Hence, released ATP, mediated by erythrocyte deoxygenation, will be effectively transferred to purinergic receptors on the endothelium. Many studies have shown that the capillary endothelium is conductive when locally stimulated by vasodilators (Dietrich, 1989; Dietrich and Tyml, 1992a,b; Song and Tyml, 1993; Collins et al., 1998; Bagher and Segal, 2011). Therefore, we hypothesize that the capillary bed is the major site for O_2_ regulation in the microcirculation (Ellis et al., 2012).

**Figure 1:**
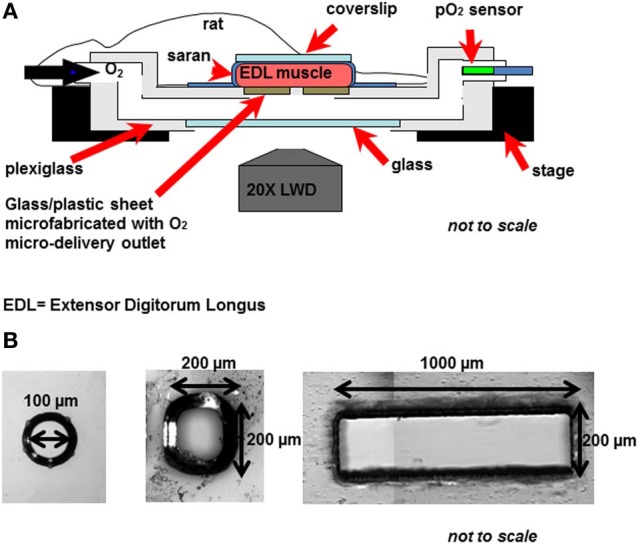
**(A)** The novel O_2_ micro-delivery approach. Extensor Digitorum Longus (EDL) rat muscle is surgically exposed and positioned on the viewing platform of an inverted microscope. O_2_ is delivered to the surface of the muscle through a micro-outlet patterned in ultrathin glass/plastic sheet (Ghonaim et al., 2011). O_2_ levels in the gas exchange chamber near the muscle surface are oscillated using computer controlled flow meters. Real-time videos of the trans-illuminated tissue are monitored and recorded using a dual-wavelength video microscopy system (Ellis et al., 1990, 1992; Japee et al., 2004, 2005a,b) **(B)** Three designs of the oxygen micro-delivery outlet are tested: circular micro-outlet (~100 μm in diameter) (Ghonaim et al., 2011), a square micro-slit (200 × 200 μ m), and a rectangular micro-slit (1000 μm wide × 200 μm long).

To test this hypothesis, we have been examining the micro-vascular response to local perturbations in tissue O_2_ tension (PO_2_) using a novel O_2_ micro-delivery tool (Ghonaim et al., 2011). We have created an O_2_ micro-delivery (and removal) system that allows for altering local tissue PO_2_ and hence erythrocyte SO_2_ in a few selected capillaries at the surface of the Extensor Digitorum Longus (EDL) muscle of the rat (Figure 1). This system replaces the gas exchange chamber originally used in our group to alter surface tissue PO_2_ of the entire bottom surface of the muscle (Ghonaim et al., 2011; Ellis et al., 2012). The chamber is positioned in the platform of an inverted microscope and is connected to computer controlled gas flow meters which allows for capturing video images of the microvascular response to PO_2_ perturbations while simultaneously controlling chamber PO_2_ levels. Erythrocyte SO_2_ values are calculated based on a dual-wavelength image capture system and video sequences are post-processed to extract functional images and hemodynamic information as previously described (Ellis et al., 1990, 1992; Japee et al., 2004, 2005a,b).

In our novel O_2_ micro-delivery setup, ultrathin plastic/glass sheet patterned with an O_2_ delivery micro-outlet replaces the gas permeable membrane in the original chamber (Ghonaim et al., 2011; Ellis et al., 2012). Data presented earlier (Ghonaim et al., 2011) show that circular micro-delivery outlets (100 μm in diameter) can alter SO_2_ in single capillaries flowing directly over the outlet. However, in order to elicit microvascular responses, the optimal outlet dimensions should allow for a sufficient number of capillaries within a network to be stimulated to produce a large enough ATP signal. This should be accomplished while ensuring the high localization of the stimulus to affect only the desired capillaries. This requires testing with various O_2_ outlet sizes and dimensions. Combining the possible technical challenges involved in creating multiple designs of the O_2_ micro-delivery device with the inherent complexities of the O_2_ regulation system led us to develop a computational model for the system under investigation.

Recently, Goldman et al. (2012) presented a theoretical mathematical model based on previous work by Goldman and Popel (1999) and Arciero et al. (2008) to describe O_2_ and ATP transport in the rat EDL microcirculation when using the original O_2_ exchange chamber. In this study we employ the same approach to calculate SO_2_ and ATP changes in selected capillaries flowing over an O_2_ delivery outlet of specific dimensions. Three designs of the O_2_ delivery micro-outlet were tested: circular outlet (100 μm in diameter), square outlet (200 × 200 μ m), and rectangular slit (200 μm long × 1000 μm wide). Average capillary SO_2_ and ATP level at steady-state were calculated at various chamber PO_2_ levels (15, 40, and 150 mmHg) relative to a zero flux boundary condition. In order to simplify the system under investigation, an idealized three dimensional (3D) parallel array capillary geometry has been used. Simulations were also run on a 3D idealized array geometry in which a terminal arteriole (9 μm in diameter) replaced 4 capillaries and was positioned 30 μm from the bottom tissue surface. These simulations allowed for investigating the potential role of the terminal arteriole in O_2_ regulation. Confirming previous findings (Ghonaim et al., 2011), the results indicated that radial O_2_ diffusion from an O_2_ delivery micro-outlet regardless of its dimensions is limited to ~50 μm, while axial diffusion affects ~100 μm of tissue. The rectangular slit has the important property of ensuring that capillaries surrounding the network of interest are all experiencing the same PO_2_ drop, which minimizes re-oxygenation and emphasizes the ATP signal. This design also produces sufficient ATP release in multiple capillaries that it should be able to consistently elicit micro-vascular responses, although this remains to be confirmed experimentally. The results presented here also predict minimal contribution of terminal arterioles to the net magnitude of ATP emerging from capillary network although they would participate as O_2_ sources and hence influence the O_2_ distribution. In the future, 3D capillary networks reconstructed from experimental data can be modeled which will provide more realistic data and help more closely predict changes in various parameters.

## Materials and methods

### Oxygen transport model

In this work, O_2_ transport and ATP transport were modeled in an idealized 3D capillary network consisting of an array of parallel capillaries (oriented in the *y* direction). The computational model of O_2_ transport was based on a finite-difference model described by Goldman and Popel (1999, 2000, 2001). In the model, the reaction-diffusion equation below was used to describe time-dependent tissue PO_2_ *P*(*x,y,z,t*):

(1) $$\frac{\partial P}{\partial t}={\left[1+\frac{c_{Mb}}{\alpha }\frac{dS_{Mb}}{dP}\right]}^{-1}\{D\nabla^{2}P-\frac{1}{\alpha }M\left(P\right)+\frac{1}{\alpha }D_{Mb}c_{Mb}\nabla \cdot \left(\frac{dS_{Mb}}{dP}\nabla P\right)\}$$

where *D* is the tissue O_2_ diffusion coefficient, α is the tissue O_2_ solubility, and *M*(*P*) is consumption rate of O_2_ in tissue (Table 1). O_2_ transport in tissue was facilitated by the presence of myoglobin where *c*_Mb_ is myoglobin concentration, *D*_Mb_ is the myoglobin diffusion coefficient, and *S*_Mb_(*P*)= *P*/(*P* + *P*_50_,_*Mb*_) is the myoglobin SO_2_. Convective transport of O_2_ in the micro-vessels at each axial location *y* was described using the following time-dependent mass balance equation for capillary SO_2_, *S*(*y,t*):

(2) $$\frac{\partial S}{\partial t}=-{\left[C+\alpha_{b}\frac{dP_{b}}{dS}\right]}^{-1}\left\{-u\left[\tilde{C}+{\tilde{\alpha }}_{b}\frac{dP_{b}}{dS}\right]\frac{\partial S}{\partial y}-\frac{1}{\pi R}\oint j\cdot d\theta \right\}$$

where *u* is the mean blood velocity, *R* is the capillary radius, *j* is the O_2_ flux at (*y*,θ) out of the capillary, *C* and $\tilde{C}$ are blood O_2_-binding capacities, respectively, directly related to hematocrit:

$$\begin{array}{l} C=H_{T}C_{\text{Hb}}\\ \tilde{C}=H_{D}C_{\text{Hb}} \end{array}$$

where *H*_*T*_ is tube (volume-weighted) hematocrit, *H*_*D*_ is discharge (flow-averaged) hematocrit, and *C*_Hb_ is the binding capacity of hemoglobin (Table 1) (Goldman and Popel, 2001). *P*_b_ is the blood PO_2_, and α_*b*_ and ${\tilde{\alpha }}_{b}$ are volume- and flow-weighted blood O_2_ solubilities, respectively (Goldman and Popel, 2001), where,

$$\begin{array}{l} \alpha_{b}=H_{T}\alpha_{\text{cell}}+\left(1-H_{T}\right)\alpha_{\text{pl}}\\ \;\tilde{\alpha }=H_{D}\alpha_{\text{cell}}+\left(\text{1}-H_{D}\right)\alpha_{\text{pl}} \end{array}$$

where α_cell_ and α_pl_ are the O_2_ solubilities inside the erythrocyte and in the plasma (Goldman and Popel, 2001). The O_2_ flux at the capillary-tissue interface was given by:

(3) $$j=\kappa (P_{b}-P_{w})$$

where κ is the mass transfer coefficient and *P*_w_ is the tissue PO_2_ at the capillary surface. κ is a function of hematocrit as it describes the effect of RBC spacing on O_2_ diffusion and exchange between capillary and tissue (Eggleton et al., 2000). At the capillary surface, the boundary condition was specified as:

(4) $$-D\alpha \frac{\partial P_{w}}{\partial n}=j$$

where *n* is the unit vector normal to the capillary surface and *j* is given by equation (3). In the model presented here, zero O_2_ flux conditions (no O_2_ exchange across tissue boundary) were specified at the tissue boundaries, except where PO_2_ was fixed on part or all of the bottom surface to represent the effect of the O_2_ exchange chamber (see below). As in the model described by Goldman and Popel (1999), Michaelis–Menten consumption kinetics, *M* = *M*_0_*P*/(*P* + *P*_cr_), and the Hill equation for the oxyhemoglobin saturation curve, *S*(*P*) = *P*^*n*^/(*P*^*n*^ + *P*^*n*^_50_), were used along with the above O_2_ transport equations to calculate tissue O_2_ transport.

**Table 1:** **Parameter values used in oxygen and ATP transport simulations**.

**Parameter**	**Value**
α	3.89 × 10^−5^ ml O_2_ ml^−1^ mmHg^−1^
*D*	2.41 × 10^−5^ cm^2^ s^−1^
*P*_cr_	0.5 mmHg
*c*_Mb_	1.02 × 10^−2^ ml O_2_ ml^−1^
*D*_Mb_	3 × 10^−7^ cm^2^ s^−1^
*P*_50_	37 mmHg
*n*(Hill exponent)	2.7
*P*_50, Mb_	5.3 mmHg
*C*_Hb_	0.52 ml O_2_ ml^−1^
*v*_rbc_	1.45 × 10^−2^ cm s^−1^
*H*_T_	0.19
*H*_D_	0.2
*C*_0_	1.4 × 10^−9^ mol s^−1^· cm^−3^
*C*_1_	0.891
*k*_d_	2.0 × 10^−4^ cm s^−1^

**Figure 2:**
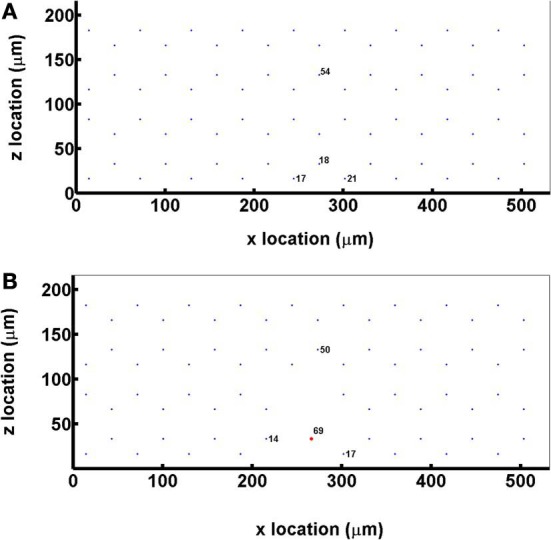
**(A)** A cross sectional view of the idealized capillary parallel array geometry showing the positioning and numbering of the 72 hexagonally arranged capillaries in the modeled network. **(B)** A cross sectional view of the idealized capillary parallel array geometry with a terminal arteriole (vessel 69, 9 μm in diameter) replacing 4 capillaries within 30 μm from bottom tissue surface.

Hemodynamic parameters (erythrocyte mean velocity, *v*_rbc_, and hematocrit, *H*_T_) were determined from *in vivo* experimental measurements in the EDL muscle of the rat. The capillary network consisted of 72 parallel capillaries, each of which was discretized into 50 cylindrical segments, and the tissue domain surrounding the capillaries had dimensions of 216 × 532 × 500 μm and was discretized into 7,304,853 computational nodes using a grid spacing of approximately 2 μm (Figure 2A). Capillary entrance SO_2_ (65%) and the tissue O_2_ consumption rate (1.5 × 10^−4^ ml O_2_/ml/s) were set based on previous experimental data (Fraser et al., 2012).

For simulations that included a terminal arteriole in the 3D network geometry, the arteriole (9 μm in diameter) was positioned ~30 μm from the bottom tissue surface and replaced 4 capillaries in the original parallel array capillary geometry (Figure 2B). Simulations including the arteriole were run at both 65 and 80% arteriolar entrance SO_2_.

### ATP transport model

ATP transport in the idealized 3D capillary network was modeled as described by Goldman et al. (2012), based on the O_2_ transport mathematical model described above (Goldman and Popel, 2000). Using a capillary entrance ATP concentration of zero, plasma [ATP] was calculated by using a finite-difference method to solve the following continuum partial differential equation (Goldman et al., 2012):

(5) $$\left(1-H_{T}\right)\frac{\partial }{\partial t}\left[\text{ATP}\right]=-u\left(1-H_{D}\right)\frac{\partial }{\partial y}\left[\text{ATP}\right]+H_{T}C_{0}\left(1-C_{1}S\right)-\frac{2}{R}k_{d}\left[\text{ATP}\right]$$

where *u* is the mean blood velocity at axial location *y*, *H*_*D*_, and *H*_*T*_ are the discharge and tube hematocrit, respectively, and *R* is capillary radius. *C*_0_ and *C*_1_ (Table 1) are constants used to linearly approximate the ATP release rate as a function of SO_2,_ while *k*_*d*_ provides an approximation of ATP degradation by the endothelium as previously described (Arciero et al., 2008).

To calculate the steady-state distributions of tissue PO_2_ and capillary SO_2_ and [ATP], time-dependent O_2_ transport and ATP transport simulations were run, using zero initial conditions for all variables, until there were minimal changes in tissue O_2_ consumption and PO_2_, and capillary O_2_ flux, SO_2_ and [ATP] between consecutive time steps.

### Tissue PO_2_ boundary conditions used to model oxygen exchange chamber

For the idealized capillary geometry, 3D tissue PO_2_ distribution and capillary [ATP] at steady state were calculated for O_2_ delivery using full gas exchange chamber, circular micro-outlet (100 μm in diameter), square micro-outlet (200 × 200 μm), or a rectangular micro-slit (1000 μm wide × 200 μm long). For each chamber type, simulations were run at 3 PO_2_ boundary conditions either over full surface (with full gas exchange chamber) or only at the micro-slit opening: 15, 40, and 150 mmHg. For the cases in which the PO_2_ boundary condition is altered only at the microslit opening, the rest of the tissue surrounding the micro-slit is set to zero O_2_ flux boundary condition. The results from all simulations were compared to a fourth control case in which full surface is set to zero O_2_ flux boundary condition.

For the idealized capillary geometry that includes the terminal arteriole, O_2_ diffusion was modeled for full chamber or a rectangular micro-slit (1000 μm wide × 200 μm long) at the 4 PO_2_ boundary conditions discussed above. Each set of simulations was run with arteriolar entrance SO_2_ of 65% or to 80%. Table 2 lists the summary of simulations and boundary conditions used in this study.

**Table 2:** **List of boundary conditions used in oxygen and ATP transport simulations**.^**^

**Network specifications**	**Chamber type tested**	**Corresponding figure in manuscript**	**PO_2_ condition at chamber outlet (in each chamber type tested)**
Capillary array	Full chamber	3	• Zero O_2_ flux (Control)
	Circle	4	• 40 mmHg
	Square	5	• 15 mmHg
	Rectangle	6	• 150 mmHg
Capillary array with arteriole (entrance SO_2_ = 65%)	Full chamber	8	• Zero O_2_ flux (Control)
			• 40 mmHg
	Rectangle	10	• 15 mmHg
			• 150 mmHg
Capillary array with arteriole (entrance SO_2_ = 80%)	Full chamber	9	• Zero O_2_ flux (Control)
			• 40 mmHg
	Rectangle	11	• 15 mmHg
			• 150 mmHg

** Summary of transport simulations, chamber types, and boundary conditions.

## Results

### Mathematical modeling of SO_2_-dependent ATP release in capillary networks in response to localized tissue PO_2_ perturbations

In this study, the release of ATP in capillaries mediated by tissue hypoxia and the de-saturation of hemoglobin was modeled in a 3D idealized parallel capillary network. The dependence of the magnitude of total ATP release on the number of de-oxygenated capillaries was also examined. Based on our previously described experimental work (Ghonaim et al., 2011), we mathematically simulated O_2_ delivery to and removal from selected capillaries on the surface of skeletal muscle tissue (rat EDL) using three designs of O_2_ exchange micro-outlets used in our *in vivo* experiments (Figure 1). In order to compare local O_2_ perturbations using the micro-outlets to global perturbations using the full gas exchange chamber (Ghonaim et al., 2011; Ellis et al., 2012), O_2_ delivery to and removal from the entire bottom tissue surface was also modeled. For each set of simulations, 3D tissue PO_2_ distribution profiles and corresponding 3D capillary [ATP] maps were generated. Plots of calculated SO_2_ and [ATP] along the length of selected capillaries (21, 18, 17, 54) at steady state were also created. All simulations were run using software written in Fortran, and the results were analyzed and the plots were produced using MATLAB.

#### Full surface gas exchange chamber

**Figure 3:**
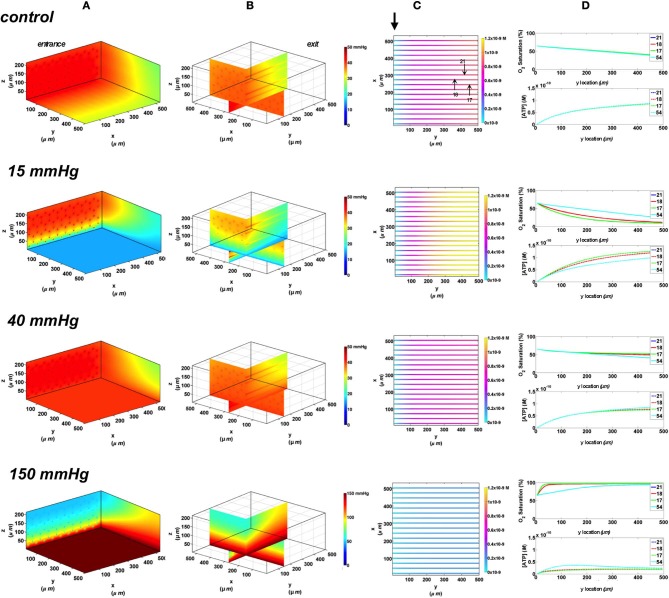
**Simulations of 3D PO_2_ and capillary [ATP] distribution in a tissue with idealized parallel capillary arrangement (72 hexagonally packed capillaries).** In these simulations, we are modeling O_2_ delivery to bottom tissue surface using the full gas exchange chamber (Ghonaim et al., 2011; Ellis et al., 2012). Full bottom tissue surface is exposed to 15, 40, or 150 mmHg chamber PO_2_ level relative to a zero flux control boundary condition **(A)** Spatial 3D tissue PO_2_ distribution (mmHg) at capillary entrance perspective **(B)** a capillary exit perspective showing combined *X–Z* plane slice at *Y* = 150 μm and *Y–Z* plane slice at *X* = 277 μm **(C)** bottom perspective of a vessel map depicting distribution of [ATP] (mol/L = M) along the capillaries. Bolded arrow marks capillary entrance **(D)** Plots of SO_2_ (%) and [ATP] changes along capillary length in selected capillaries (21, 18, 17, 54) marked by arrows on the vessel map. Capillaries 21 and 17 are 16 μm from tissue surface, capillary 18 is 33 μm from tissue surface, and capillary 54 is deeper in the tissue, at 133 μm, and hence it is not shown in the current perspective of the vessel map. Note change in PO_2_ scale from 0 to 50 mmHg in first three cases to 0–150 mmHg when surface is exposed to 150 mmHg.

In this set of simulations, the 3D PO_2_ distribution in the tissue and corresponding SO_2_ and [ATP] distribution along capillary length were modeled for the control scenario in which the full bottom tissue surface is exposed to PO_2_ perturbations. This would experimentally simulate using the full gas exchange chamber. As shown in Figure 3, at 40 mmHg, steady-state tissue PO_2_ and capillary [ATP] distributions are comparable to the no-flux control condition. At the venular end of the capillaries, SO_2_ values ranged from ~50% for surface capillaries (21, 18, and 17) to ~40% for capillaries deeper than 100 μm into the tissue (capillary 54), and the corresponding capillary [ATP] values were within 15% of those at zero O_2_ flux boundary condition. However, under imposed tissue hypoxia (15 mmHg), the surface capillaries dropped their SO_2_ by ~70% which corresponded to ~40% higher steady state capillary [ATP] relative to zero flux condition (Figure 3C). This was clearly depicted in the corresponding vessel map (Figure 3B). The deeper capillary (54) was less affected with ~30% lower hemoglobin SO_2_ and ~12% increase in ATP release relative to zero flux. Exposing the full tissue surface to relatively high chamber PO_2_ (150 mmHg) had the most significant impact on [ATP] in the capillary network. At 150 mmHg, hemoglobin SO_2_ in both surface and deep tissue capillaries converged to ~100% with ~70% decrease in steady state [ATP] relative to no-flux (Figure 3C). The depth of the PO_2_ perturbation into the tissue when using the full gas exchange chamber was ~100 μm as shown in the 3D PO_2_ profiles (Figure 3A).

#### Circular O_2_ delivery micro-outlet

**Figure 4:**
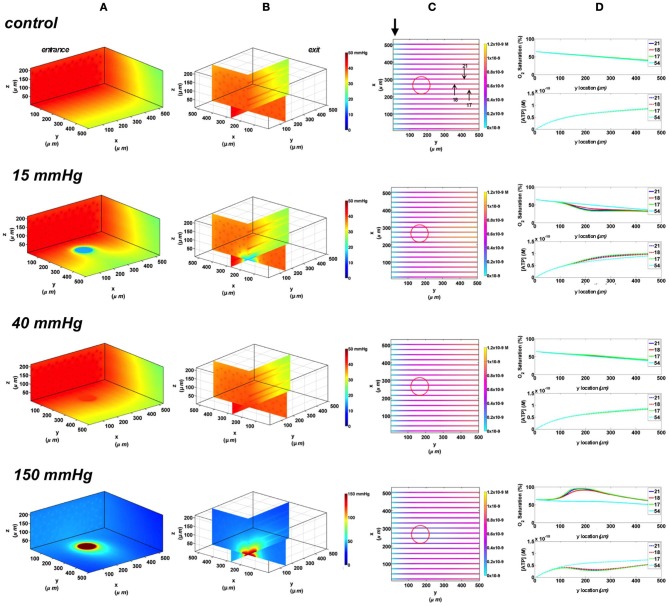
**Simulations of 3D PO_2_ and capillary [ATP] distribution in a tissue with idealized parallel capillary arrangement (72 hexagonally packed capillaries).** In these simulations, we are modeling O_2_ delivery through a circular oxygen micro-delivery outlet (100 μm in diameter) to bottom tissue surface using novel micro-delivery approach (see Figure 1). Tissue surface directly on top of the micro-delivery outlet is exposed to 15, 40, or 150 mmHg chamber PO_2_ level relative to a zero flux control boundary condition **(A)** Spatial 3D tissue PO_2_ distribution (mmHg) at capillary entrance perspective **(B)** a capillary exit perspective showing combined *X–Z* plane slice at *Y* = 150 μm and *Y–Z* plane slice at *X* = 277 μm **(C)** bottom perspective of a vessel map depicting distribution of [ATP] (mol/L = M) along the capillaries. Bolded arrow marks capillary entrance **(D)** Plots of SO_2_ (%) and [ATP] changes along capillary length in selected capillaries (21, 18, 17, 54) marked by arrows on the vessel map. Capillaries 21 and 17 are 16 μm from tissue surface, capillary 18 is 33 μm from tissue surface, and capillary 54 is deeper in the tissue, at 133 μm, and hence it is not shown in the current perspective of the vessel map.

To investigate the effect of limiting the number of capillaries stimulated by local tissue PO_2_ perturbations, we started by modeling capillary SO_2_ and [ATP] changes when using a circular O_2_ micro-outlet (100 μm in diameter, see Figure 1). Similar to previously discussed data (Ghonaim et al., 2011), substantive changes in local tissue PO_2_ due to diffusion outwards from the circular outlet is limited to less than ~50 μm, as shown in the 3D tissue PO_2_ profiles (Figure 4A). Also, the hypoxic and hyperoxic stimuli were highly localized to only those capillaries directly over the micro-outlet (17, 18, 21) as shown in the vessel maps (Figure 4B). At 40 mmHg chamber PO_2_ level, calculated capillary SO_2_ and [ATP] were in close agreement with the no-flux control for both surface and deep tissue capillaries with values being within ~1 and ~3%, respectively (Figure 4C). Under imposed hypoxia, the capillary SO_2_ dropped as capillaries crossed the micro-outlet region reaching a minimum value ~40 μm downstream of the outlet after which SO_2_ levels increased slightly due to re-oxygenation by surrounding capillaries. At the venular end, steady state SO_2_ levels in surface capillaries were 15% lower relative to zero flux while capillary 54 experienced only a 6% drop in SO_2_. This corresponded to only 10% increase in [ATP] in surface capillaries while [ATP] in capillary 54 remained unchanged relative to zero flux. At 150 mmHg, the increase in capillary SO_2_ level is observed directly over the micro-outlet region reaching a maximum at the outlet exit. The capillary SO_2_ levels dropped sharply downstream of the outlet due to O_2_ diffusion into adjacent capillaries and tissue. Surface capillary SO_2_ decreased to ~63 and deep tissue capillaries to 51% ~200 μm downstream of the outlet. This corresponded to ~40% decrease in [ATP] in surface capillaries and ~20% decrease in [ATP] of deeper tissue capillaries relative to zero flux condition.

#### Square O_2_ delivery micro-outlet

**Figure 5:**
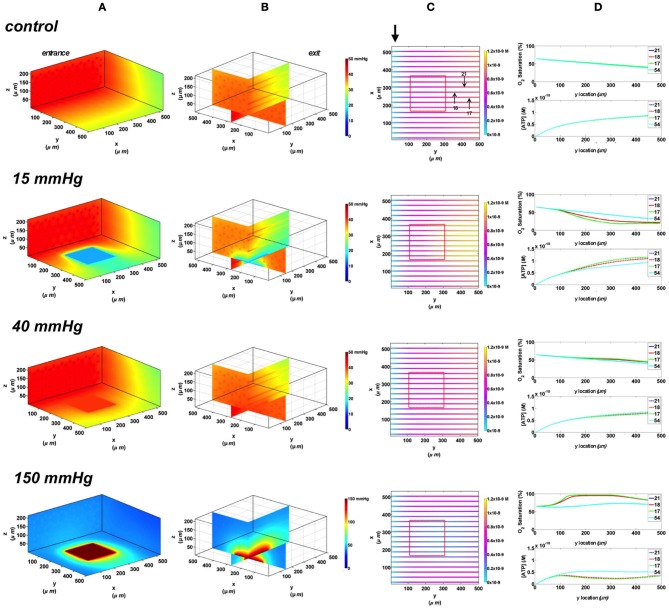
**Simulations of 3D PO_2_ and capillary [ATP] distribution in a tissue with idealized parallel capillary arrangement (72 hexagonally packed capillaries).** In these simulations, we are modeling O_2_ delivery through a square oxygen micro-delivery outlet (200 × 200 μm) to bottom tissue surface using our previously described novel micro-delivery approach (see Figure 1). Tissue surface directly on top of the micro-delivery outlet is exposed to 15, 40, or 150 mmHg chamber PO_2_ level relative to a zero flux control boundary condition **(A)** Spatial 3D tissue PO_2_ distribution (mmHg) at capillary entrance perspective **(B)** a capillary exit perspective showing combined *X–Z* plane slice at *Y* = 150 μm and *Y–Z* plane slice at *X* = 277 μm **(C)** bottom perspective of a vessel map depicting distribution of [ATP] (mol/L = M) along the capillaries. Bolded arrow marks capillary entrance **(D)** Plots of SO_2_ (%) and [ATP] changes along capillary length in selected capillaries (21, 18, 17, 54) marked by arrows on the vessel map. Capillaries 21 and 17 are 16 μm from tissue surface, capillary 18 is 33 μm from tissue surface, and capillary 54 is deeper in the tissue, at 133 μm, and hence it is not shown in the current perspective of the vessel map.

Next, we simulated the effect of increasing the area of O_2_ exchange, and hence perturbing a greater number of capillaries, by simulating an O_2_ delivery micro-outlet 200 × 200 μm square. Similar to the circular micro-outlet design and as previously described (Ghonaim et al., 2011), the change of local tissue PO_2_ surrounding the square outlet is limited to less than ~50 μm, as shown in the 3D tissue PO_2_ profiles (Figure 5A). In the case of the square micro-outlet, a larger number of surface capillaries experience the PO_2_ perturbations, 7 of which were directly over the micro-outlet (Figure 5B). Also, capillaries at both sides of those directly over the outlet seemed to be slightly affected by the PO_2_ perturbations. At 40 mmHg, calculated SO_2_ and capillary [ATP] distributions were similar to the no-flux control with surface capillaries having 15% higher SO_2_ and 10% lower [ATP] values relative to zero flux condition (Figure 5C). As observed with the circular micro-outlet, re-oxygenation of stimulated capillaries following imposed hypoxia (15 mmHg) was at ~40 μm downstream of the square micro-outlet (Figure 5C). At the capillary venular end, SO_2_ level of surface capillaries dropped by ~51% while capillary 54 experienced only a 15% drop in SO_2_ relative to zero flux. This corresponded to ~32% increase in [ATP] in surface capillaries while only 7% increase in [ATP] in capillary 54 relative to zero flux. At 150 mmHg, capillary SO_2_ levels increased across the micro-delivery outlet reaching maximum values at the venular end of the outlet region. Similar to the results observed with the circular micro-outlet, SO_2_ values sharply dropped downstream of the square micro-outlet bringing surface capillary SO_2_ to ~83% and deeper capillaries to ~70% ~200 μm downstream of the outlet. This corresponded to ~66% decrease in [ATP] in surface capillaries and ~42% decrease in [ATP] of deeper tissue capillaries.

#### Rectangular O_2_ delivery micro-slit

**Figure 6:**
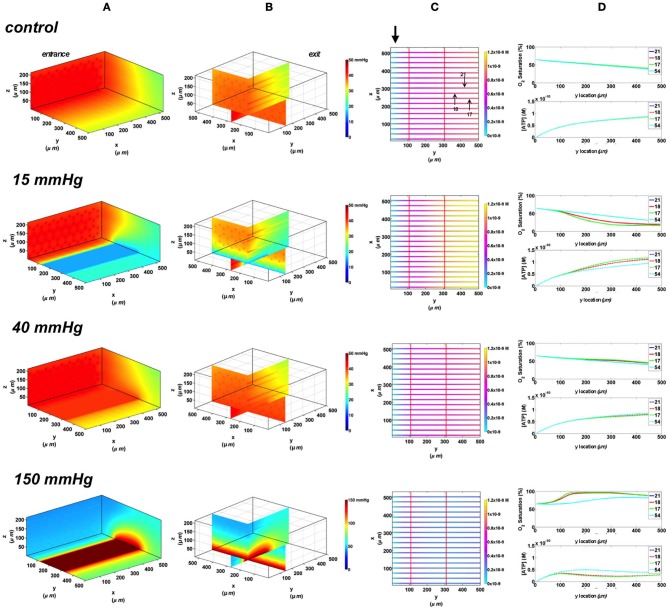
**Simulations of 3D PO_2_ and capillary [ATP] distribution in a tissue with idealized parallel capillary arrangement (72 hexagonally packed capillaries).** In these simulations, we are modeling O_2_ delivery through a rectangular oxygen micro-delivery outlet (1000 μm wide × 200 μm long) to bottom tissue surface using our previously described novel micro-delivery approach (see Figure 1). Tissue surface directly on top of the micro-delivery outlet is exposed to 15, 40, or 150 mmHg chamber PO_2_ level relative to a zero flux control boundary condition **(A)** Spatial 3D tissue PO_2_ distribution (mmHg) at capillary entrance perspective **(B)** a capillary exit perspective showing combined *X–Z* plane slice at *Y* = 150 μm and *Y–Z* plane slice at *X* = 277 μm **(C)** bottom perspective of a vessel map depicting distribution of [ATP] (mol/L = M) along the capillaries. Bolded arrow marks capillary entrance **(D)** Plots of SO_2_ (%) and [ATP] changes along capillary length in selected capillaries (21, 18, 17, 54) marked by arrows on the vessel map. Capillaries 21 and 17 are 16 μm from tissue surface, capillary 18 is 33 μm from tissue surface, and capillary 54 is deeper in the tissue, at 133 μm, and hence it is not shown in the current perspective of the vessel map.

The largest dimensions for an O_2_ delivery micro-outlet currently being tested in our *in vivo* studies are for a rectangular micro-slit (1000 μm wide × 200 μm long). Since the 3D tissue dimensions in our computational model are less than the width of the experimental micro-slit, the effect of the slit extends to both edges of the tissue allowing us to visualize the depth of the PO_2_ distribution into the tissue. As shown in the 3D PO_2_ plots (Figure 6A), the PO_2_ perturbations extended ~100 μm into the tissue with local tissue PO_2_ changes similar to what was observed with other outlet designs. All surface capillaries shown on the vessel map are affected by the PO_2_ perturbation as the outlet covers the entire surface width (Figure 6B). At 40 mmHg, calculated SO_2_ and capillary [ATP] distributions were similar to the no-flux control with surface capillaries having 17% higher SO_2_ and 10.3% lower [ATP] values relative to zero flux O_2_ boundary condition (Figure 6C). Under imposed hypoxia (15 mmHg), re-oxygenation of de-saturated surface capillaries was not observed within 200 μm downstream of the micro-slit. However, capillary SO_2_ seemed to plateau approximately 100 μm downstream of the micro-slit. At the capillary venular end, SO_2_ level of surface capillaries dropped by ~56% while capillary 54 experienced a ~20% drop in SO_2_ relative to zero flux condition. This corresponded to ~35% increase in [ATP] in surface capillaries and only 8% increase in [ATP] in capillary 54 relative to zero flux. At 150 mmHg, capillary SO_2_ levels increased across the micro-delivery outlet reaching maximum values at the venular end of the outlet region. Similar to the results observed with the previously discussed micro-outlet designs, SO_2_ values instantly dropped downstream of the rectangular micro-slit bringing surface capillary SO_2_ to ~90% and deep capillaries to ~80% ~200 μm downstream of the outlet. This corresponded to ~69% decrease in [ATP] in surface capillaries and ~55% decrease in [ATP] of deeper tissue capillaries.

#### Comparing change in relative ATP magnitude in response to varying the area of imposed tissue hypoxia

**Figure 7:**
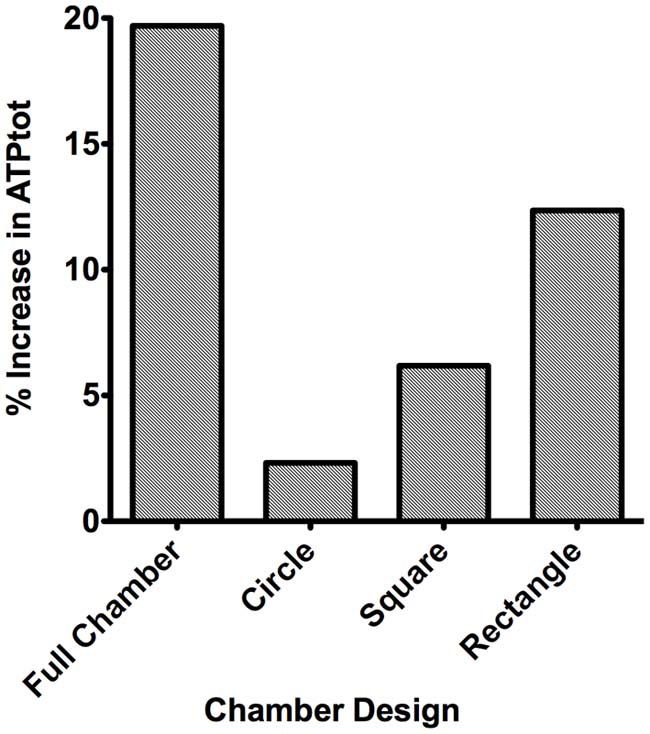
**Percent increase in the total magnitude of ATP (ATPtot) relative to zero flux control boundary condition calculated for idealized parallel capillary network with no arteriole at 15 mmHg chamber PO_2_ level.** Percent increase in ATP magnitude in the network were calculated when entire bottom tissue surface is exposed to the PO_2_ perturbation using full gas exchange chamber or locally using a circular (100 μm in diameter), square (200 × 200 μm) or rectangular O_2_ delivery micro-slit (1000 μm wide × 200 μm long).

The change in the total magnitude of ATP (ATPtot) in the modeled network relative when imposing a hypoxic challenge (15 mmHg boundary condition) was calculated as percent increase above ATPtot at zero flux (Figure 7). Percent increase in ATP magnitude in the network was compared when exposing all of the bottom tissue surface to hypoxia (full chamber) or locally using the three micro-outlet designs discussed above. As shown in Figure 7, the total ATP magnitude increased with increase in micro-outlet dimensions and essentially the number of capillaries experiencing the PO_2_ drop. The percent increase in ATPtot was more than doubled when locally perturbing tissue PO_2_ using the rectangular micro-slit compared to the other micro-outlet designs. The total ATP magnitude calculated when limiting the area of tissue hypoxia using the rectangular micro-slit was only 38% lower relative to full exposed surface (Figure 7). The increase in the total ATP magnitude in a network exposed to local hypoxia was minimal (~2%) when using the circular micro-outlet or and only 6% above that zero flux when using the square micro-outlet.

### Mathematical modeling of arteriolar SO_2_ and ATP concentration in response to localized tissue PO_2_ perturbations

In order to investigate the role terminal arterioles play in regulating SO_2_-mediated ATP signaling in capillary networks, particularly in the EDL muscle where larger arterioles are located much deeper in the tissue, the 3D idealized capillary geometry was modified to include a terminal arteriole, 9 μm in diameter, positioned 30 μm away from bottom tissue surface. The 3D tissue PO_2_ distribution as well as SO_2_ and [ATP] in the arteriole (vessel 69) and in the surrounding surface (capillaries 14 and 17) and deep tissue capillaries (represented by capillary 50) were modeled. Simulations were run for the case in which the full tissue surface is exposed to PO_2_ perturbations (original gas exchange chamber) and for the case of spatially limited O_2_ delivery using the rectangular O_2_ delivery micro-slit. Also, the effect of varying arteriolar entrance SO_2_ on the overall magnitude of ATP in response to altered tissue PO_2_ was examined.

#### Full surface gas exchange chamber at 65 and 80% arteriolar entrance SO_2_

**Figure 8:**
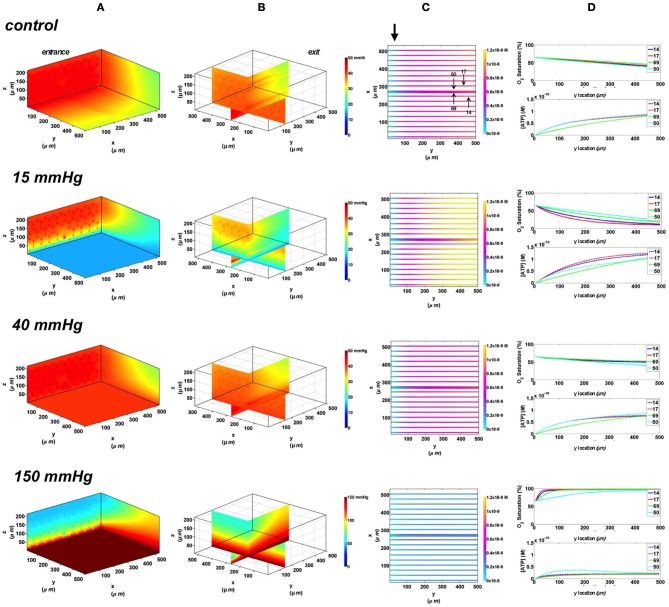
**Simulations of 3D PO_2_ and capillary [ATP] distribution in a tissue with idealized parallel capillary arrangement (68 hexagonally packed capillaries), which includes a traversing terminal arteriole (vessel 69) at an entrance SO_2_ of 65%.** In these simulations, we are modeling O_2_ delivery to bottom tissue surface using the full gas exchange chamber (Ghonaim et al., 2011; Ellis et al., 2012). Full bottom tissue surface is exposed to 15, 40, or 150 mmHg chamber PO_2_ level relative to a zero flux control boundary condition **(A)** Spatial 3D tissue PO_2_ distribution (mmHg) at capillary entrance perspective **(B)** a capillary exit perspective showing combined *X–Z* plane slice at *Y* = 150 μm and *Y–Z* plane slice at *X* = 266 μm **(C)** bottom perspective of a vessel map depicting distribution of [ATP] (mol/L = M) along the arteriole and surrounding capillaries. Bolded arrow marks arteriolar and capillary entrance **(D)** Plots of SO_2_ (%) and [ATP] changes along vessel length in selected vessels (14, 17, 69-arteriole, 50) marked by arrows on the vessel map. Capillary 17 is 16 μm from tissue surface, the arteriole and capillary 14 are 33 μm from tissue surface, and capillary 50 is deeper in the tissue, at 133 μm, yet is shown adjacent to the arteriole in the current perspective of the vessel map.

**Figure 9:**
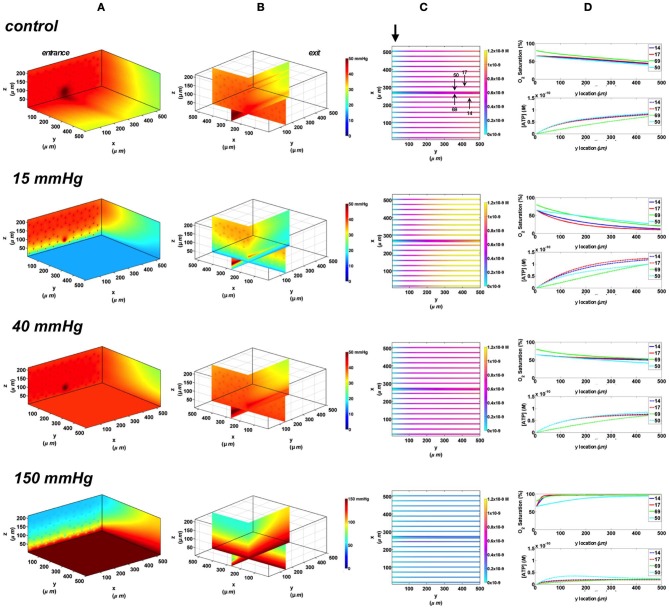
**Simulations of 3D PO_2_ and capillary [ATP] distribution in a tissue with idealized parallel capillary arrangement (68 hexagonally packed capillaries), which includes a traversing terminal arteriole (vessel 69) at an entrance SO_2_ of 80%.** In these simulations, we are modeling O_2_ delivery to bottom tissue surface using the full gas exchange chamber (Ghonaim et al., 2011; Ellis et al., 2012). Full bottom tissue surface is exposed to 15, 40, or 150 mmHg chamber PO_2_ level relative to a zero flux control boundary condition **(A)** Spatial 3D tissue PO_2_ distribution (mmHg) at capillary entrance perspective **(B)** a capillary exit perspective showing combined *X–Z* plane slice at *Y* = 150 μm and *Y–Z* plane slice at *X* = 266 μm **(C)** bottom perspective of a vessel map depicting distribution of [ATP] (mol/L = M) along the arteriole and surrounding capillaries. Bolded arrow marks arteriolar and capillary entrance **(D)** Plots of SO_2_ (%) and [ATP] changes along vessel length in selected vessels (14, 17, 69-arteriole, 50) marked by arrows on the vessel map. Capillary 17 is 16 μm from tissue surface, the arteriole and capillary 14 are 33 μm from tissue surface, and capillary 50 is deeper in the tissue, at 133 μm, yet is shown adjacent to the arteriole in the current perspective of the vessel map.

In the 3D tissue PO_2_ profiles and [ATP] vessel maps generated for these simulations, the PO_2_ perturbations were shown to affect the terminal arteriole to a much lesser extent than the surface capillaries (Figures 8A,B, 9A,B). Also, these simulations showed the influence of the arteriole as an O_2_ source on the SO_2_ levels of nearby surface capillaries. For instance, the steady state SO_2_ level in capillary 14, positioned right next to the arteriole, was ~25% higher than the zero flux control condition when exposed to 40 mmHg chamber PO_2_ and identical to the SO_2_ level of the terminal arteriole (Figures 8, 9). However, the SO_2_ level of the deeper tissue capillary (50), which was located at the same depth as capillary 54, was unchanged relative to zero flux. In general, the different arteriolar entrance SO_2_ has no effect on the surface or deep tissue capillaries and had minimal influence on the arteriolar SO_2_ at steady state. At 15 mmHg, the SO_2_ level of the terminal arteriole entering at 65% dropped by 60% relative to zero flux condition corresponding to 44% increases in [ATP]. A smaller drop in SO_2_ was calculated (52% decrease) for the arteriole entering at 80% corresponding to 40% increase in [ATP]. The SO_2_ level in the surrounding surface capillaries as well as deeper tissue capillaries dropped by ~70 and 35%, respectively, corresponding to ~45 and 16% higher steady state capillary [ATP] relative to zero flux (Figures 8C, 9C). At 150 mmHg, hemoglobin SO_2_ levels in surface and deep tissue capillaries as well as in the arteriole converged to ~100% with ~77% decrease in steady state [ATP] in the capillaries and 75% decrease in [ATP] in the arteriole relative to zero flux control (Figures 8C, 9C).

#### Rectangular oxygen delivery micro-slit at 65 and 80% arteriolar entrance SO_2_

**Figure 10:**
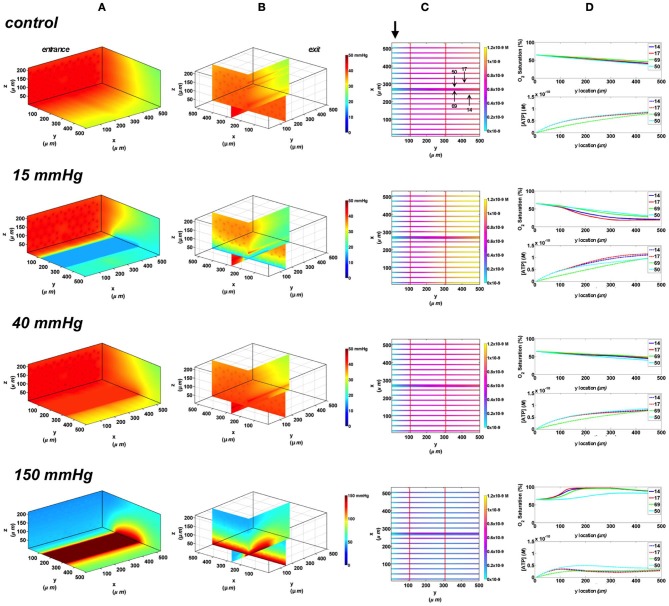
**Simulations of 3D PO_2_ and capillary [ATP] distribution in a tissue with idealized parallel capillary arrangement (68 hexagonally packed capillaries), which includes a traversing terminal arteriole (vessel 69) at an entrance SO_2_ of 65%.** In these simulations, we are modeling O_2_ delivery through a rectangular oxygen micro-delivery outlet (1000 μm wide × 200 μm long) to bottom tissue surface using our previously described novel micro-delivery approach (see Figure 1). Tissue surface directly on top of the micro-delivery outlet is exposed to 15, 40, or 150 mmHg chamber PO_2_ level relative to a zero flux control boundary condition **(A)** Spatial 3D tissue PO_2_ distribution (mmHg) at capillary entrance perspective **(B)** a capillary exit perspective showing combined *X–Z* plane slice at *Y* = 150 μm and *Y–Z* plane slice at *X* = 266 μm **(C)** bottom perspective of a vessel map depicting distribution of [ATP] (mol/L = M) along the arteriole and surrounding capillaries. Bolded arrow marks arteriolar and capillary entrance **(D)** Plots of SO_2_ (%) and [ATP] changes along vessel length in selected vessels (14, 17, 69-arteriole, 50) marked by arrows on the vessel map. Capillary 17 is 16 μm from tissue surface, the arteriole and capillary 14 are 33 μm from tissue surface, and capillary 50 is deeper in the tissue, at 133 μm, yet is shown adjacent to the arteriole in the current perspective of the vessel map.

**Figure 11:**
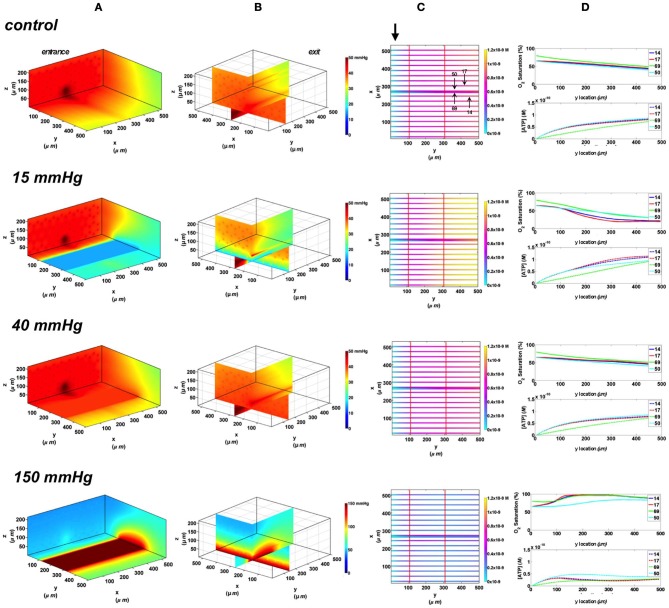
**Simulations of 3D PO_2_ and capillary [ATP] distribution in a tissue with idealized parallel capillary arrangement (68 hexagonally packed capillaries), which includes a traversing terminal arteriole (vessel 69) at an entrance SO_2_ of 80%.** In these simulations, we are modeling O_2_ delivery through a rectangular oxygen micro-delivery outlet (1000 μm wide × 200 μm long) to bottom tissue surface using our previously described novel micro-delivery approach (see Figure 1). Tissue surface directly on top of the micro-delivery outlet is exposed to 15, 40, or 150 mmHg chamber PO_2_ level relative to a zero flux control boundary condition **(A)** Spatial 3D tissue PO_2_ distribution (mmHg) at capillary entrance perspective **(B)** a capillary exit perspective showing combined *X–Z* plane slice at *Y* = 150 μm and *Y–Z* plane slice at *X* = 266 μm **(C)** bottom perspective of a vessel map depicting distribution of [ATP] (mol/L = M) along the arteriole and surrounding capillaries. Bolded arrow marks arteriolar and capillary entrance **(D)** Plots of SO_2_ (%) and [ATP] changes along vessel length in selected vessels (14, 17, 69-arteriole, 50) marked by arrows on the vessel map. Capillary 17 is 16 μm from tissue surface, the arteriole and capillary 14 are 33 μm from tissue surface, and capillary 50 is deeper in the tissue, at 133 μm, yet is shown adjacent to the arteriole in the current perspective of the vessel map.

In these simulations, the capillary array that includes the terminal arteriole is exposed to local perturbations in tissue PO_2_ through the rectangular micro-slit. At 40 mmHg chamber PO_2,_ the calculated steady state SO_2_ and [ATP] levels at the venular end of surface and deep tissue capillaries as well as in the arteriole are within 5% of those at zero flux condition and uninfluenced by the arteriolar entrance SO_2_ (Figures 10, 11). Under imposed hypoxia (15 mmHg), the calculated arteriolar SO_2_ values at steady state were 50% higher than the case in which the full surface is exposed to the PO_2_ perturbations and identical to those of deeper tissue capillaries. Hence, a minimal drop in SO_2_ (38% decrease) was calculated in the arteriole relative to zero flux. These arteriolar steady state SO_2_ values were uninfluenced by the different arteriolar entrance SO_2_. The influence of the arteriole as an O_2_ source to nearby capillaries downstream of the micro-slit can be clearly observed in the 3D PO_2_ profiles at 15 mmHg (Figures 10A, 11A). However, the surface capillaries (14, 17) experienced a sharper drop in SO_2_ in response to the imposed hypoxia with 53% drop in SO_2_ and a corresponding 39% increase in [ATP]. As observed when locally stimulating surface capillaries in the absence of the arteriole, capillaries were re-oxygenated ~40 μm downstream of the hypoxic micro-slit region. At 150 mmHg, the steady state SO_2_ levels in surface capillaries and in the arteriole converged to ~88% while the SO_2_ level of capillary 50 was slightly lower at 83% which corresponded to 65% and 57% decrease in [ATP], respectively, relative to zero flux (Figures 10C, 11C).

#### Estimating relative arteriolar ATP magnitude in response to tissue PO_2_ perturbations

**Figure 12:**
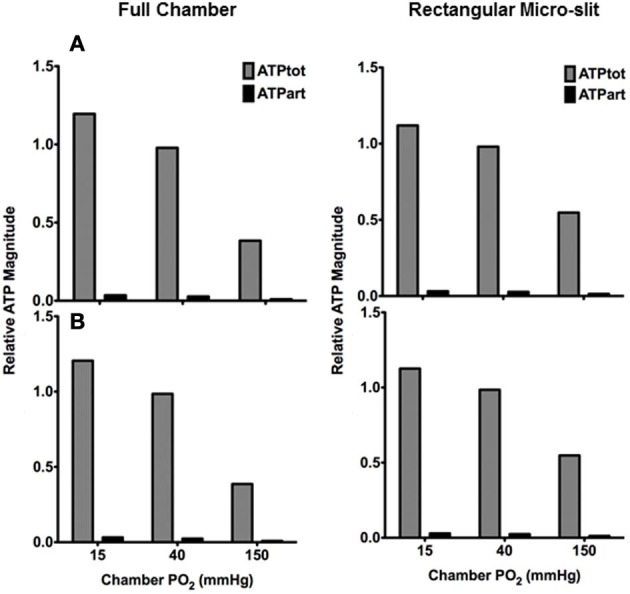
**Total ATP magnitude (moles) at steady state calculated for entire network (ATPtot from all 68 capillaries) or in the terminal arteriole only (ATPart) normalized against ATPtot calculated at no-flux condition.** Relative ATP magnitudes are calculated for an idealized 3D parallel capillary array network with a terminal arteriole (9 μm in diameter) positioned 30 μm from tissue surface. Simulations were run with entire bottom tissue surface being exposed to PO_2_ perturbations using full gas exchange chamber or locally using a rectangular O_2_ delivery micro-slit (1000 μm wide × 200 μm long). For both conditions, relative ATP magnitudes are calculated for the case in which the terminal arteriole has an entrance SO_2_ of **(A)** 65% or **(B)** 80%.

In order to estimate the contribution of the terminal arteriole to ATP mediated signaling in capillary networks, the steady state magnitude of ATP in the arteriole (ATPart) at various tissue PO_2_ conditions was calculated and normalized against total ATP magnitude in the network (ATPtot) under zero flux condition (Figure 12). The relative arteriolar ATP magnitudes were calculated when full tissue surface is exposed to the PO_2_ perturbations (full gas exchange chamber) or to local perturbations using the rectangular O_2_ delivery micro-slit. As shown in Figure 12, the arteriolar ATP magnitude decreased with increase in chamber PO_2_ level. However, the model suggested that under hypoxic conditions (15 mmHg), the terminal arteriole would contribute less than 3% of the total ATP signal originating from a capillary network. Also, although the percent decrease in ATP magnitude in the arteriole is similar to that calculated for the total network when increasing chamber PO_2_ from 15 to 150 mmHg, the absolute change in ATP magnitude (moles) in the arteriole is ~95% less. Finally, it should be noted that [ATP] in the arteriole is ~20% lower when limiting area of PO_2_ perturbations using the rectangular micro-slit.

## Discussion

In the microcirculation, ATP is released from the erythrocytes in an SO_2_ dependent manner. Released ATP would bind to purinergic receptors on the vascular endothelium which activates a signaling pathway leading to the opening of Ca^2+^ gated K^+^ channels and the hyperpolarization of the endothelial cell (Ellsworth et al., 2008; Tran et al., 2012). The hyperpolarization signal is then conducted upstream through gap junctions. At the arteriolar wall, the incoming hyperpolarization signal is conducted to the SMC layer through myo-endothelial gap junctions resulting in vaso-relaxation and increase in erythrocyte supply rate (Ellsworth et al., 2008; Tran et al., 2012). The magnitude of the hyperpolarization signal would depend on the number of endothelial cells activated along the capillary and on the total number of capillaries stimulated within a network under hypoxic conditions. This understanding of how erythrocyte-released ATP controls micro-vascular O_2_ delivery is consistent with the modeling results presented in this paper. The net increase in total ATP magnitude in the network with increase in the area exposed to hypoxia is the summative contribution of additional stimulated capillaries (Figures 3–7). Also, these results help explain our observations of no vascular response when experimentally testing the effect of O_2_ delivery through a circular micro-outlet (100 μm in diameter) *in vivo* (Ghonaim et al., 2011). Although this design maybe optimal for locally altering SO_2_ in single capillaries, the stimulus would probably not be sufficient to elicit a micro-vascular response. Increasing the dimensions of the micro-outlet would be necessary to stimulate a large enough number of capillaries, thus amplifying total magnitude of ATP release and signal.

Also, as our modeling data suggest, increasing the micro-outlet dimensions minimizes the effect of stimulated capillary re-oxygenation downstream of the micro-outlet. This is because the capillaries of interest would be surrounded by capillaries experiencing the same drop in PO_2_. This is more representative of the situation *in vivo* as the outlet physiologically simulates an arteriole crossing the capillary bed acting as an O_2_ source or a venule withdrawing O_2_, which would affect multiple capillaries. In terms of the signaling response, delayed re-oxygenation following hypoxic stimulation ensures the ATP signal persists longer distances downstream thus stimulating a larger number of endothelial cells. Since each endothelial cell in skeletal muscle is ~100 μm long, using the rectangular slit is estimated to activate at least 3.5 endothelial cells in each stimulated capillary. In comparison with the square micro-outlet, which has the same length (200 μm) as the rectangular micro-slit, ~1 more endothelial cell is activated per capillary with the latter design. It should be noted that in the modeled geometry, which lacks realistic capillary branching and has an idealized, uniform capillary density, we are examining relative changes in the total magnitude of ATP when using various outlet designs. During *in vivo* experiments, a maximum of two micro-vascular units ~10 capillaries may be positioned along the entire width of the rectangular micro-slit, while only one or two capillaries with a branching point could be positioned over the circular micro-outlet (Ghonaim et al., 2011). Hence a 1000 μm wide × 200 μm long outlet might cover the threshold number of capillaries needed to elicit a micro-vascular response. This indicates the rectangular micro-slit would be optimal for stimulating enough capillaries by imposed hypoxia to generate high enough ATP signal.

The limited amount of change in tissue PO_2_ due to diffusion (~50 μm), as measured from the 3D tissue PO_2_ profiles, beyond the edge of the micro-outlets (Figures 4A–6A and 4B–6B) was consistent with our previous observations (Ghonaim et al., 2011). The simulations indicated that the PO_2_ perturbations are highly localized to only those capillaries directly over the micro-outlet region. Experimentally, the results suggest that the micro-outlet should be positioned at least 50 μm downstream of the terminal feeding arteriole to ensure that micro-vascular responses are only elicited from the capillaries positioned directly over the outlet. The extent of axial O_2_ diffusion in the tissue when using the rectangular micro-slit was 50% deeper than that previously modeled for the circular micro-outlet (Figures 6B, 10B, 11B) (Ghonaim et al., 2011) and similar to that of the full surface model (Ghonaim et al., 2011; Ellis et al., 2012). Due to the shape of the PO_2_ profile, the maximal axial diffusion distance is estimated from the center of the outlet. The increase in the axial diffusion distance might be a compromise when using larger O_2_ micro-outlets. With our current microscopic techniques we are unable to resolve vessels deeper than 60 μm.

Since in our experiments, we examine micro-vascular signaling from selected capillaries, it was critical that we assess the possible contribution of arterioles beyond our ability to focus. Since arterioles have relatively higher erythrocyte velocities than in the capillaries, they are anticipated to experience a much lesser change in SO_2_ in response to PO_2_ perturbations. This was supported by our simulation data (Figures 8–11). The main effect of a nearby terminal arteriole on a capillary within 50 μm is that it would act as an O_2_ source. As shown in our modeling data (Figures 8D–11D), higher measured SO_2_ in a capillary relative to other capillaries with comparable flow rates in the same preparation might imply the presence of a nearby arteriole. Since arterioles in the EDL muscle preparation are deeper in the tissue, their contribution to the total magnitude of ATP in a locally stimulated capillary network is probably negligible. The contribution of a terminal arteriole positioned directly over the micro-slit ~30 μm from bottom surface was calculated to be less than 3% of the total magnitude of the ATP (Figure 12). Hence, when locally stimulating capillaries, even in the presence of an underlying arteriole, the observed micro-vascular responses mediated by intra-luminal ATP would be primarily due to ATP released in the stimulated capillaries.

In conclusion, we have modeled SO_2_-dependant changes in [ATP] at steady state in 3D idealized parallel capillary networks in response to local PO_2_ perturbations. As the number of affected capillaries increases, the total magnitude ATP in the network increases. The results indicated that O_2_ delivery or removal to overlaying tissue through a rectangular micro-slit (1000 μm wide × 200 μm long) would be optimal relative to other micro-outlet designs of smaller dimensions or a full surface classical exchange type chamber. Using the rectangular micro-slit it is anticipated that a sufficient number of capillaries will be stimulated to produce a large enough magnitude of ATP to elicit micro-vascular responses. This would be accomplished while maintaining the stimulus localized to the selected capillaries. The results also indicated that terminal arterioles have minimal influence on the total magnitude of ATP in the network under hypoxic condition. Hence, when locally stimulating the capillary bed, the majority of the signal elicited by ATP release would originate in the capillaries. The computational model presented provides valuable insights into how to study the ATP release mechanism and signaling in capillary networks *in vivo*. The modeling data help guide us in the design of an optimal tool for studying SO_2_-dependent ATP release in capillaries *in vivo*. In the future, we aim to model time-dependent ATP release to local PO_2_ perturbations in a realistic capillary network geometry reconstructed from experimental data. Combining our *in vivo* experimental observations with computational modeling of the dynamics of SO_2_-dependent ATP release will help provide a more comprehensive understanding of O_2_ mediated blood flow regulation in micro-vascular networks.

### Conflict of interest statement

The authors declare that the research was conducted in the absence of any commercial or financial relationships that could be construed as a potential conflict of interest.
